# Tinnitus Associated with Mild Osteomyelitis of the Temporal Bone Reversed after Conservative Antibiotic Treatment: A Case Series

**DOI:** 10.3390/medicina58020318

**Published:** 2022-02-20

**Authors:** Ping-Tao Tseng, Tien-Yu Chen, Chun-Chung Lui, Yen-Wen Chen, Jiann-Jy Chen

**Affiliations:** 1 Prospect Clinic for Otorhinolaryngology & Neurology, Kaohsiung 811, Taiwan; kevinachen0527@gmail.com; 2 Department of Psychology, College of Medical and Health Science, Asia University, Taichung 413, Taiwan; 3 Institute of Biomedical Sciences, National Sun Yat-sen University, Kaohsiung 804, Taiwan; 4 Department of Psychiatry, Tri-Service General Hospital, School of Medicine, National Defense Medical Center, Taipei 114, Taiwan; verducciwol@gmail.com; 5 Institute of Brain Science, National Yang Ming Chiao Tung University, Taipei 112, Taiwan; 6 Division of Medical Image, Department of Radiology, E-Da Cancer Hospital, Kaohsiung 824, Taiwan; lchung3@gmail.com; 7 Department of Otorhinolaryngology, E-Da Cancer Hospital, Kaohsiung 824, Taiwan

**Keywords:** tinnitus, osteomyelitis, antibiotic, HRCT, computed tomography

## Abstract

The symptomatology and diagnostic tools for osteomyelitis of the temporal bone have not been well documented. Diagnosis of early stage (i.e., mild form) osteomyelitis of the temporal bone may be delayed due to the limitations of traditional computed tomography’s (CT) imaging resolution. With the advancement of high-resolution CT (HRCT) images, clinicians can now observe images that could not be observed with traditional CT imaging. In this neuro-image report, we present three patients with refractory/untreatable tinnitus. In their HRCT images, mild osteomyelitis of the temporal bone was revealed by mucosa thickening with bony sequestration of air cells, mild opacification of the air cells, and soft tissue density in the middle ear cavity, mild opacification, and bony sequestration attributed to mucosa thickening of the mastoid air cells (along with the cortical bone). All of the clinical presentations and findings in the pure tone audiometry of the reported patients improved after adequate antibiotic treatment. The current report highlights the potential benefit of HRCT to diagnose this in such patients. In addition, immediate and conservative antibiotic treatment is recommended for managing these patients shortly after the detection of mild osteomyelitis of the temporal bone. This treatment could reduce the risk of progression to the severe form.

## 1. Letter

Tinnitus is a subjective discomfort with a presentation of annoying noise within unilateral/bilateral ears. Among the adult population, the prevalence of tinnitus is as high as 20% [1]. There has not yet been consistent evidence concerning the etiology of tinnitus. Several hypotheses, including local infection/inflammation in the inner ear [2], oxidative stress imbalance [3], and abnormalities in the dopaminergic system [4] have been suggested. Due to its unknown etiology or origin, the diagnosis of “tinnitus” is difficult and mainly based on subjective symptoms. Although pure tone audiometry (PTA) might be helpful in the evaluation of tinnitus severity, it is neither specific nor particularly sensitive to tinnitus. Therefore, tinnitus in most patients is considered to be “primary” or “without specific origin” and empirically treated. Several pharmacologic or non-pharmacologic interventions with various mechanism have been developed, contributed to various effective treatments [5,6].

Osteomyelitis of the temporal bone is rare. It could manifest in a mild or severe form [7]. In its severe form, the mortality rate could be as high as 46% in the early stages. This is referred to as malignant otitis externa and requires surgical intervention [7]. Due to its rare prevalence, the symptomatology and diagnostic tools for this condition have not been well documented. Due to the limitations of traditional computed tomography’s (CT) imaging resolution, early stage (i.e., mild form) osteomyelitis of the temporal bone may not be detected, leading to a delayed diagnosis. Currently, with the development of new technologies and the progress of computer programs, CT resolution has been improved. With the advancement of high-resolution CT (HRCT) (i.e., 0.5 mm/slice) images, clinicians can now observe images that could not be observed with traditional CT imaging. In this neuro-image report, we present the HRCT images of patients with mild osteomyelitis of the temporal bone. These patients initially presented with refractory/untreatable tinnitus.

**Figure 1:**
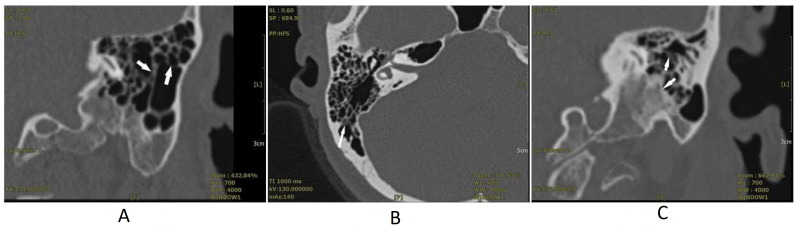
High-resolution computed tomography (HRCT) results. The white arrow indicates the following: mucosa thickening and bony sequestration of air cells in the coronal view of Mr. H’s HRCT examination (**A**), mild opacification of the air cells along with the cortical bone and bony sequestration in the axial view of Ms. R’s HRCT examination (**B**), and soft tissue density in the middle ear cavity, mild opacification and bony sequestration of the mastoid air cells along with the cortical bone in the coronal view of Mrs. C’s HRCT examination (**C**).

**Figure 2:**
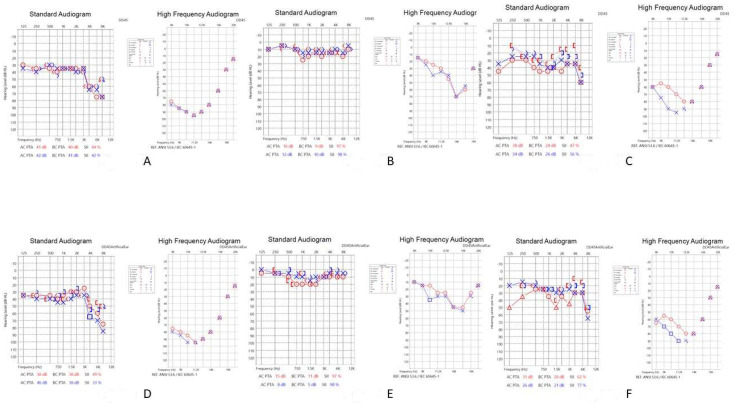
Depiction of the baseline (**A**–**C**) and follow-up (**D**–**F**) pure tone audiometry (PTA) results. In the PTA, the Right ear is present in Red color, while the Left ear is present in Blue color. In general, the PTA revealed mild improvements in the standard audiogram and high-frequency audiogram in the right ear and the standard audiogram of the left ear of Mr. H (**A**,**D**). In case of Ms. R it showed mild improvements in the standard audiogram and high-frequency audiogram of the left ear (**B**,**E**). It also revealed improvements in the standard audiogram and high-frequency audiogram of the left ear and the standard audiogram of the right ear in case of Ms. C (**C**,**F**). O: right ear unmasked air conduction; △: right ear masked air conduction; X: left ear unmasked air conduction; □: left ear unmasked air conduction; < >: right/left unmasked bone conduction; [ ]: right/left masked air conduction.

Mr. H was a 61-year-old driver and had a past history of bilateral tinnitus (left side louder than the right side) for eight years. He was treated with anxiolytics due to tinnitus-related insomnia and had a past history of hypertension, hyperlipidemia, and fatty liver. He had received general laboratory survey and ordinary brain CT without abnormal findings. This patient visited our clinic in September 2020 with baseline tinnitus handicap inventory (THI) [8] scores of 74. He was referred to receive HRCT to rule out a potential intra-cranial lesion associated with his chronic tinnitus. HRCT without contrast revealed mucosa thickening with bony sequestration of air cells (Figure 1A). The baseline PTA conducted in September 2020 revealed mild symmetrical high tone sensorineural hearing loss (Figure 2A). There was no abnormal finding noted in the other tests. The patient was diagnosed with a mild form of osteomyelitis of the temporal bone and consequent tinnitus, and we initiated treatment with intravenous ceftriaxone 1 gm once every week for 10 months. His tinnitus and hearing improved after one-year of treatment with follow-up THI scores of 36. He received a follow-up PTA in December 2021. This revealed mild improvements in the standard audiogram and high-frequency audiogram of the right ear and standard audiogram of the left ear (Figure 2D). Even after discontinuation of the medication, his symptoms continued to improve.

Ms. R was a 46-year-old tradesperson without any history of systemic disease. She began to have occasional bilateral tinnitus after she gave birth to her first child. The tinnitus gradually got worse and disturbed her daily life, resulting in severe anxiety, which prompted her to visit our clinic. Her blood laboratory examination revealed insignificant findings. The baseline THI scores were 76. We referred her for an HRCT to rule out potential intra-cranial lesions. The HRCT without contrast revealed mild opacification of the air cells along with cortical bone and bony sequestration (Figure 1B). The baseline PTA in August 2020 revealed symmetrically super-high-tone sensorineural hearing loss (Figure 2B). No abnormal findings were noted in the other test. Under the impression that the patient was experiencing a mild form of osteomyelitis of the temporal bone and consequent tinnitus, we initiated treatment with an antihistamine and intravenous ceftriaxone 1 gm once every two weeks for one year. In addition, to treat her anxiety, we prescribed a low dose of a selective serotonin reuptake inhibitor. Her tinnitus and sleep quality improved after 1-year of treatment. The follow-up PTA in December 2021 revealed mild improvements in the standard audiogram and high-frequency audiogram of the left ear (Figure 2E). The post-treatment THI scores were 34.

Ms. C was a 65-year-old patient with a five-year history of dull tinnitus in her left ear. She reported that the dull tinnitus had worsened and was accompanied with hyperacusis during the past one to two years. She had a past history of hypertension, diabetes mellitus, and a small old infarct over the left frontal lobe. The other past ordinary brain CT and blood laboratory examination did not reveal any significant findings. She visited our clinic due to hyperacusis. Her THI scores before treatment were 82. HRCT without contrast was arranged to rule out potential intra-cranial lesions. It revealed soft tissue density in the middle ear cavity, mild opacification, and bony sequestration attributing to mucosa thickening of the mastoid air cells along with the cortical bone (Figure 1C). The baseline PTA in February 2021 revealed symmetrically super-high-tone severe sensorineural hearing loss and generalized mild conductive hearing loss (Figure 2C). There were no abnormal findings noted in the other test. Under the impression that the patient was experiencing a mild form of osteomyelitis of the temporal bone and consequent tinnitus, we initiated an antihistamine and intravenous ceftriaxone 1 gm once per three days treatment for six months and then changed to Q2W for the next six months. Her hyperacusis and dull tinnitus improved. The follow-up PTA in December 2021 revealed improvements in the standard audiogram and high-frequency audiogram of left ear and standard audiogram of the right ear (Figure 2E). The THI scores in the follow-up period were 38.

## 2. Discussion

To our knowledge, this is the first report addressing reversible tinnitus associated with a mild form of osteomyelitis of the temporal bone, which responded adequately to conservative antibiotic treatment. Further, certain patients with impaired hearing ability experienced improved PTA findings after antibiotic treatment. The potential differential diagnosis of these three cases according to their history included labyrinthitis with inflammatory tinnitus, auditory hallucination with tinnitus, and senori-neuro-conductive hearing loss with compensatory tinnitus. However, auditory hallucinations with tinnitus were less likely after the psychiatrist’s evaluation. There was no other evidence to approve the existence of labyrinthitis with inflammatory tinnitus. Finally, senori-neuro-conductive hearing loss with compensatory tinnitus was less likely improved by antibiotics treatment. From the view of neuro-image, there were several differential diagnoses to be addressed, such as osteoma, fibrousdysplasia, and abscess. However, these diseases would not be relieved by simple and low-dosage ceftriaxone treatment. We chose PTA rather than HRCT as the follow-up test since PTA is comparatively less invasive and expensive. As for the choice of image diagnostic tool, we chose HRCT but not magnetic resonance imaging (MRI) due to the unfavorably expensive cost of MRI. However, in a situation of contraindication to HRCT (i.e., pregnancy), we might have no other choice but to choose MRI. The diagnosis of osteomyelitis of the temporal bone was based on the neuroimage findings in the HRCT. This included soft tissue density in the middle ear cavity and opacification and bony sequestration was attributed to mucosa thickening of the mastoid air cells (along with the cortical bone) [7]. Although the association between osteomyelitis of the temporal bone and tinnitus remains unclear, previous reports found that tinnitus might be associated with tumor invasion [9] or inflammation [10] of the temporal bone. In the present cases, testing for the potential origins of tinnitus revealed insignificant findings. We choose to use low-dosage and conservative ceftriaxone treatment since there were no any danger signs or red flag in these three patients, which indicated less necessity for high-dosage or advance antibiotics treatment. The treatment focused on the mild form osteomyelitis of the temporal bone and resulted in tinnitus improvement, which might reflect the potential association between osteomyelitis of the temporal bone and tinnitus. We aimed to highlight the potentially mild form of osteomyelitis of the temporal bone in patients with refractory/untreatable tinnitus and benefit of HRCT to diagnose this condition in these patients. In addition, immediate and conservative antibiotic treatment is recommended for managing these patients shortly after the detection of mild osteomyelitis of the temporal bone. This treatment could reduce the risk of progression to more severe forms of this condition.

## Data Availability

The data of the current report was available upon reasonable request.
